# University Student Depression Inventory, Brazilian Version, Construct Assessment

**DOI:** 10.1590/0034-7167-2023-0232

**Published:** 2024-07-29

**Authors:** Fernanda Pâmela Machado, Marcos Hirata Soares, Katya Luciane de Oliveira, Regina Celia Bueno Rezende Machado, Adriano Luiz da Costa Farinasso, Margarita Antonia Villar Luís

**Affiliations:** I Universidade Estadual de Londrina. Londrina, Paraná, Brazil; II Escola de Enfermagem de Ribeirão Preto. Ribeirão Preto. São Paulo, Brazil

**Keywords:** Validation Study, Factor Analysis, Statistical, Depression, Students, Patient Health Questionnaire, Estudio de Validación, Análisis Factorial, Depresión, Estudiantes, Cuestionario de Salud del Paciente

## Abstract

**Objectives:**

to assess the University Student Depression Inventory, Brazilian version (USDI-BR), construct.

**Methods:**

a methodological study carried out with a snowball probabilistic sample, consisting of 334 undergraduate and graduate students. Confirmatory factor analysis, reliability using McDonald’s omega coefficient and Cronbach’s alpha were performed. Principal component analysis was performed using the varimax rotation and oblimin rotation, using the Kaiser-Meyer-Olkin criteria, Bartlett’s test of sphericity and scree plot.

**Results:**

the USDI-BR presented an internal consistency of items of ω = 0.95 and remained with 30 items, with the addition of 1 factor (Death wish and social withdrawal), totaling 4 factors.

**Conclusions:**

the USDI-BR has evidence that points to its validity and also its internal consistency, deserving that new studies be carried out to expand the evidence of its psychometric properties.

## RESUMEN

**Objetivos:**

evaluar el constructo del *University Student Depression Inventory*, versión brasileña (USDI-BR).

**Métodos:**

estudio metodológico, realizado con una muestra probabilística en bola de nieve compuesta por 334 estudiantes de pregrado y posgrado. Se realizaron análisis factoriales confirmatorios, confiabilidad mediante el coeficiente omega de McDonald y alfa de Cronbach. El análisis de componentes principales se realizó mediante rotación ortogonal varimax y rotación oblicua oblimin, utilizando los criterios de Kaiser-Meyer-Olkin, la prueba de esfericidad de Bartlett y *scree plot*.

**Resultados:**

el USDI-BR presentó una consistencia interna de ítems de ω = 0,95 y quedó con 30 ítems, con la adición de 1 factor (Deseo de muerte y alejamiento social), totalizando 4 factores.

**Conclusiones:**

el USDI-BR tiene evidencias que apuntan a su validez y también a su consistencia interna, ameritando que se realicen nuevos estudios para ampliar la evidencia de sus propiedades psicométricas.

## INTRODUCTION

Depression is a common mental disorder around the world. As it is a disease with a significant increase in recent years, the World Health Organization (WHO) points out that, by 2030, it will be more prevalent across the planet^(1-2)^.

Due to its “common” occurrence, many researchers carry out studies around the world seeking actions to minimize this problem. Two studies highlight that depression has four main characteristics: cognitive symptoms; physiological symptoms; behavioral symptoms; and motivational symptoms^(3-4)^.

These symptoms can affect people with depression, but in young adults, they intensify due to the changes they are subjected to, especially those in the academic phase. Various situations, such as external demands, self-demand, physiological and hormonal changes, social and political changes, social class, dissatisfaction and overload, can be identified in young adults in the academic phase^(1,5)^.

In addition to these changes, which compromise academic and personal performance, there is concern about suicidal behaviors. Currently, a young adult who presents depressive symptoms is at greater risk of suicidal behavior than other people. This risk in this population is related to the domains of this research, with academic motivation and/or its absence, the cognitive/emotional aspect and lethargy leading to social withdrawal and, consequently, desire to die^(6-7)^.

In particular, depressive symptoms associated with the changes and demands required by academic life contribute to an increase in suicidal behavior. This statement is proven through scientific studies carried out on this population in Brazil, in which young adults, undergraduate and/or graduate students present risk factors for depressive symptoms^(8-11)^.

Due to the increase in risk factors and depressive symptoms in this population, the number of university students who suffer from psychological problems is noticeable in clinical practice and literature. Thinking about this increase and, as it has not yet been identified in Brazilian literature, an instrument with satisfactory evidence of specific content validity for students, two researchers^(6)^ translated and adapted the University Student Depression Inventory, Brazilian version (USDI-BR) for Brazil, which presented satisfactory evidence of content validity^(6)^. This instrument aims to measure the level of depressive symptoms in university students. The original scale was created and validated^(4)^ by Khawaja and Bryden in 2006 at the University of Queensland.

In Brazil, translation and cross-cultural adaptation were carried out^(6)^, i.e., content validation has already been carried out. However, for this instrument to be used, construct validity is necessary.

Thinking about answering the USDI-BR construct, in this study, construct validity was carried out through the application of the translated and validated instrument in the target population divergently, analyzing whether the scale is measuring the construct efficiently through confirmatory factor analysis (CFA).

## OBJECTIVES

To assess the USDI-BR psychometric properties.

## METHODS

### Ethical aspects

The research was approved by the Research Ethics Committee in 2020, and is in accordance with Resolution 466 of 2012. All research participants received the link via Google^®^ through Google Forms^®^ and had access to a copy of the Informed Consent Form (ICF).

### Study design, place and period

This is a methodological study, carried out with undergraduate students in the state of Paraná, Brazil, from August 2021 to January 2023.

### Population, inclusion and exclusion criteria

The sample was non-probabilistic, snowball type, and consisted of 334 undergraduate students from the following areas of knowledge: exact and earth sciences; biological sciences; engineering; health sciences; linguistics; languages and arts; agricultural sciences; applied social sciences; and human sciences. The criteria for student participation in the research was to be over 18 years old and be enrolled in an undergraduate course in any area of knowledge. Students who were away for health reasons were excluded from the study.

### Study protocol

In the first stage, a brief search by title was carried out in the literature. The search was carried out in the Virtual Health Library (VHL), Scientific Electronic Library Online (SciELO) databases, following the Descriptors in Health Sciences (DeCS): “Depression”, “Validation Study”, “Students”. The filter was applied for the last five years, limited to national literature to better elucidate the topic, but no scale aimed at university students was found, only the cross-cultural adaptation study of USDI-BR^(6)^.

In the second stage, the USDI-BR, the Life Orientation Test (LOT-R)^(12)^ and the ICF were added to Google Forms^®^, following the Brazilian National Research Ethics Commission (CONEP - *Comissão Nacional de Ética em Pesquisa*) standards. The interviewed student had the option of downloading the ICF in PDF and keeping a copy as well as choosing to accept or refuse to participate in the research.

In the third stage, it was sent to students via WhatsApp^®^, Instagram^®^, Facebook^®^ groups, electronic email, and they were asked to forward it to acquaintances. This stage took place over a period of one year.

### Analysis of results, and statistics

For divergent or discriminant validation, LOT-R was used, whose objective of this measure is to assess USDI-BR divergent validity, i.e., it analyzed the difference between the USDI-BR construct and LOT-R that, theoretically, showed no correlation. Spearman’s correlation coefficient (ranks) was used. This is a non-parametric correlation^(13)^.

LOT-R measures how people perceive their lives (more optimistic or less optimistic), containing 10 items, divided into positive (items 1, 4 and 10), negative (items 3, 7 and 9) and neutral (2, 5, 6 and 8). On a 5-point Likert scale, with graduations from 0 to 4, interviewees assessed the degree of agreement or disagreement in relation to the LOT-R questions. Values were represented from 0 to 4, where: 0 = completely disagree; 1 = disagree; 2 = neutral; 3 = agree; and 4 = completely agree. In Brazil, the validation study showed satisfactory internal consistency (α =0.68).

For exploratory factor analysis (EFA) and CFA, they were entered and analyzed using the Statistical Package for the Social Sciences (SPSS) version 25.

To analyze the USDI-BR internal consistency, McDonald’s omega coefficient (ω) and Cronbach’s alpha (α) were calculated. Principal component analysis (PCA) was performed applying varimax orthogonal rotation and oblimin oblique rotation after consideration of the Kaiser-Meyer-Olkin (KMO) criteria, Bartlett’s test of sphericity (BTS) and scree plot.

## RESULTS

### Sample characterization

A total of 334 students from nine areas of knowledge participated in this study. Of these, 79.3% were female (n=265) and 20.4% were male (n=68). No student declared themselves as non-binary. Regarding age group, 39.2% were between 20 and 23 years old (n=131), 19.8%, between 24 and 27 years old (n=66), 13.8%, between 17 and 19 years old (n=46), 13.5%, > 30 years old (n=47), 10.8%, between 28 and 30 years old (n=36), 40 years old (n=1) 45 years old (n=2), 52 years old (n=1), 53 years old (n=2) and 57 years old (n=2).

### Reliability analysis

USDI-BR reliability analysis was tested using McDonald’s omega coefficient, presenting an internal consistency of items of ω:0.959 and Cronbach’s alpha of 0.957. For the LOT-R, it was also tested and presented an internal consistency of ω:0.790.

### Divergent validity

In Table 1, it can be identified, in the second column (overall LOT-R), for all domains, the correlation presenting a negative charge, suggesting that USDI-BR has divergent validity with LOT-R.

**Table 1:** Correlation matrix and divergent validity of the University Student Depression Inventory scales, Brazilian version, and the Life Orientation Test, Paraná, Brazil, 2023

Scales		Overall LOT-R	Lethargy	Cognitive/emotional	Academic motivation
Overall LOT-R	Spearman	-			
	*Ρ*	-			
Lethargy	Spearman	-0.448			
	*Ρ*	<0.001	-		
Cognitive/emotional	Spearman	-0.634	0.787	-	
	*Ρ*	<.001	<0.001		
Academic motivation	Spearman	-0.450	0.767	0.689	
	*Ρ*	<.001	<0.001	<0.001	-

After analyzing reliability and divergent validity, PCA was carried out using the KMO criterion and three main rounds, using the varimax orthogonal rotation and oblimin oblique rotation method. Commonality variance <0.3 was established to exclude the item from the instrument.

**Figure 1:**
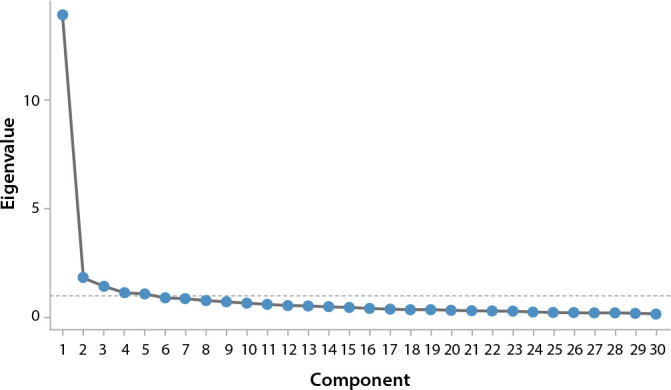
Scree plot of University Student Depression Inventory, Brazilian version, items, Paraná, Brazil, 2023

In the first round, varimax rotation and the extraction of 5 components were used. The sample initially presented a KMO of 0.950 and explained variance of 64.6%. The scree plot criterion (Figure 1) suggested the sharpest inflection point at numbers 4 and 5, suggesting a scale with 4 or 5 components.

In the second round, the extraction was of 4 components through oblimin rotation. For 4 components, there was a drop in the commonality values of 3 factors. The variance explained in this round was 61%.

In the third round, extraction was done with 3 components in oblique rotation, showing a drop in commonality values in 5 factors. When comparing oblique and varimax (orthogonal) rotations, commonality values dropped by 10 factors. Variance explained in 3 components was 57.2%. In other words, the scale would present better construct validity when arranged in 4 factors and not 3, as in its original construction.

### Confirmatory factor analysis

At this stage, factor loadings were calculated with 3 and 4 factors. As shown in Table 2, the adjustment measures were not satisfactory with 3 factors. The Corporate Adjustment Index (CAI) presented a value of 0.90 for the 4-factor index, i.e., compared to the 3-factor index, the 4-factor index appears to be a more adjusted model.

**Table 2:** Factor loadings with 3 and 4 factors of University Student Depression Inventory, Brazilian version, items, Paraná, Brazil, 2023

Factor Loadings	3 factors	4 factors
Comparative Fit Index	0.847	0.905
Tucker Lewis Index	0.834	0.893
Standardized Root Mean Square Residual	0.0552	0.0472
Root Mean Square Error of Approximation	0.0860	0.0691
Upper limit	0.0909	0.0744
Lower limit	0.0811	0.0639

In Table 3, it is possible to view the factors and descriptions as well as USDI-BR items.

**Table 3:** University Student Depression Inventory, Brazilian version, domains and item factors validated by confirmatory factor analysis, Paraná, Brazil, 2023

Factor	Description	Items (t=30)^*^
Lethargy	Characterized by a combination of physiological, behavioral and cognitive manifestations.	1,2,3,4,8,10,29,30
Cognitive/emotional	It represents cognitive symptoms, emotional symptoms.	6,11,18,20,21,22,23,24,25,26
Academic motivation	It is characterized by study-related motivation and procrastination.	7,12,13,15,16,17,19,27
Desire to die and social withdrawal	Represented by social isolation and feelings of death.	5,9,14,28

* *Total items of USDI-BR.*

Items 5 - *Eu tenho pensado em me matar*, 9 - *Eu me pergunto se vale a pena continuar vivendo*, 14 - *Passo mais tempo sozinho (a) do que costumava* and 28 - *Ninguém se importa comigo agora*, after EFA and CFA, they were added to factor 4 - Desire to die and social withdrawal (DD and SW). The definition of the name of factor 4 and the joining of these items to this factor was carried out by the authors of this study. The theoretical and statistical criteria were analyzed together to add to USDI-BR.

**Figure 2:**
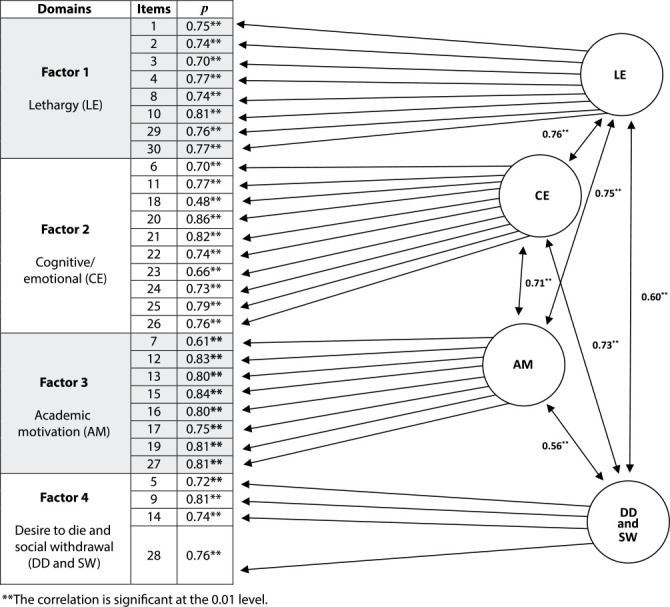
Theoretical model selected, tested and validated by confirmatory factor analysis according to the factors of the University Student Depression Inventory, Brazilian version, Paraná Brazil, 2023

Figure 2 shows the model tested by CFA. The addition of another factor is notable in the model, as had been suggested in exploratory analysis. This distribution and the addition of another factor presented significant communal values between items, different from what had been previously observed, when USDI-BR contained 3 factors.

The *p* value of items after transfer to domain 4 obtained significant results, i.e., all items, including items 5 (Ρ=0.72), 9 (Ρ=.81), 14 (Ρ=0.74) and 28 (Ρ=76)), showed a significant correlation.

In addition to the theoretical model, it is possible to analyze, through data, how related cognitive/emotional factors are to lethargy, motivation or lack of motivation in the academic context, as well as social isolation with the feeling of wanting to die among undergraduate and graduate students.

The lowest value is related to factor 2 (Item 18 - *Eu me sinto tímido(a) quando estou com outras pessoas* (*p*=0.48)). The highest value was in relation to factor 3 (item 15 - *Eu não acho os estudos tão interessantes quanto eram* (*p*=0*.84*)).

## DISCUSSION

To test an instrument’s measurement validity, studies such as those carried out by the American Educational, Research Association (AERA) and American Psychological Association (APA)^(13-14)^ consider content validity, criterion validity and construct validity as the three main techniques for this purpose.

In the USDI-BR, content validity was carried out for the Brazilian version^(6)^, presenting a total Content Validity Coefficient (tCVC) of 0.94, i.e., a value considered adequate for a measuring instrument that must be equal to or greater than 0.80^(14)^.

To validate and continue in all stages recommended in the validation process, this study carried out construct validity with a sample of 334 students. For this purpose, two coefficients were used (McDonald’s omega and Cronbach’s alpha), and both performed the same function: measuring an instrument’s reliability^(15-17)^.

Instrument reliability through McDonald’s omega analysis showed internal consistency of items of ω=0.95 and Cronbach’s alpha of α=0.95, i.e., a value that represents high reliability, as values equal to or greater than 0.70 are considered adequate in relation to the construct^(18)^. Other studies carried out with the University Student Depression Inventory (USDI) presented values close to those of the present study, such as the version in Portuguese from Portugal (α= 0.80), Persian from Iran (α= 0.94)^(19)^ and the original version, Australian English (α= 0.95)^(4)^, supporting what was identified in the USDI-BR.

For divergent validity, Spearman’s correlation was used, thereby identifying which values found in the correlation matrix (Table 1) showed negative values. The negative values presented low correlations in relation to the LOT-R and USDI-BR, i.e., LOT-R’s function is to measure expectations, optimism and future events in relation to life, presenting, in this study, low correlations in relation to all USDI-BR factors, mainly the academic motivation factor (ρ= -0.450)^(13)^.

The orthogonal rotation of factors, using the varimax method (factors are not correlated) and oblimin oblique rotation (correlated factors), as well as the KMO, BTS and scree plot criteria (Figure 1), suggested a model, *a posteriori*, where the commonality between the analyzed components suggested the addition of another factor to USDI-BR.

After the suggestive hypotheses to adapt another factor to USDI-BR, one of the tests carried out to verify the suitability of a sample is the KMO. The reference factors closest to 1 match data adjusted to factor analysis (FA)^(20-22)^. In the present study, the KMO was very significant, indicating adjusted FA (KMO= 0.95). The study carried out in Iran is similar to the value identified in this study (KMO= 0.94)^(19)^. Both studies are within the KMO classification with excellent sample suitability.

The Comparative Fit Index (CFI) in the original version, in Australian English, was > 0.90^(4)^. In the current study, the CFI with 4 factors was 0.90. and with 3 factors, 0.84. CFI compares the base model and calculates the relative fit to the model. The reference value considered “great fit to the model” is considered to be 0.9. In other words, below 0.90 indicates adequate adjustment^(21-23)^. USDI-BR is within adequate parameters.

In relation to the Tucker Lewis Index (TLI), in this study, the value was 0.83 for the 3-factor load and 0.893 for the 4-factor load, that is, they do not fit into the values considered as “great fit”, which would be 0.90 to 0.95, since TLI aims to assess the relative fits of the model when comparing it to another model^(23)^. Therefore, the adjustment in relation to TLI, in the present study, was shown to be below what is considered ideal, however a significant improvement was achieved in the 4-component model.

The Standardized Root Mean Square Residual (SRMR) also suggested that the model provided a good fit to the data (SRMR= 0.0860), and in the study carried out in Iran SRMR was 0.077^(19)^. To strengthen these data, the Root Mean Square Error of Approximation (RMSEA) must vary from 0 to 1, however values lower than or close to 0.6 suggest better adjusted models. In this study, RMSEA was tested with 3 (RMSEA= 0.0860) and 4 factors (RMSEA= 0.0691), within the limit value so that it is considered a well-adjusted model, according to this criterion.

The models tested demonstrated that the most suitable would be a scale with a factor of 4, corroborating what had already been scored by judges in the translated and adapted version of the USDI for the Brazilian version^(6)^. Therefore, USDI-BR remains with 30 items, but with 4 factors. Factor 1 refers to lethargy, 2, cognitive/emotional, 3, academic motivation, and 4, desire to die and social withdrawal. Factor 4 distributed the communal values maintaining their expected distribution. Only item 18 had a negative load.

With regard to the cognitive/emotional domain, item 18 (*Eu me sinto tímido(a) quando estou com outras pessoas*) has relatively low loadings on its factor, supporting the FA study of the original study, in which the authors of the original scale suggest that this item demonstrates a suspicious measure and should be better investigated in future studies^(4)^. This finding should be taken into consideration for the ongoing review of the psychometric properties of a measuring instrument. A scale may no longer be valid for a population due to time, cultural changes, disused terms and words, making it no longer viable for that culture.

### Study limitations

The USDI-BR showed promising results in CFA. However, it is important to note some limitations in the study. First, the number of interviewees was relatively low, which suggests the need for a larger sample that includes students from different Brazilian states, allowing for a more precise adaptation to the cultural nuances of each region. Furthermore, the predominance of female participants in the research may require adjustments to sampling design to ensure balanced representation.

### Contributions to nursing, health or public policy

The scale has good internal consistency, in addition to being, to date, the only one validated in Brazil for university students. The USDI-BR will enable healthcare professionals and professors to use an instrument that can be effective in measuring depressive symptoms in university students. That being said, it is encouraged that a scale like this would help in tracking symptoms, and with appropriate investment it could be developed to serve professionals in mental health care services.

## CONCLUSIONS

This study demonstrated, through FA, that USDI-BR presented evidence of satisfactory content validity. The 30 items were maintained, but one more factor was added, totaling 4 factors with good evidence of construct validity.

It is suggested, for future studies, to test other psychometric properties of USDI-BR, such as predictive validity and discriminant validity. It is recommended that research be carried out with undergraduate and graduate students from all over Brazil applying USDI-BR, taking into account the cultural diversity that exists in Brazil.

In addition to instrument applicability, it is recommended that they be invested in other means aimed at preventing cases (continuing education of multidisciplinary teams, health education in schools and universities), development of educational materials (e-books and folders) and dissemination on social media. In addition to these, it is important to strengthen public policies in mental health, such as housing, income, work, education and social assistance.

## References

[1] World Health Organization (WHO) (2021). Depressão.

[2] Beck AT. (1967). Depression: clinical, experimental and theoretical aspects.

[3] Khawaja NG, Bryden KJ. (2006). The development and psychometric investigation of the university student depression inventory. J Affect Disord.

[4] Aloufi MA, Jarden RJ, Gerdtz MF, Kapp S. (2021). Reducing stress, anxiety and depression in undergraduate nursing students: systematic review. Nurse Educ Today.

[5] Machado FP, Soares MH. (2022). Cross-cultural adaptation of the University Student Depression Inventory for Brazil. Rev Bras Enferm.

[6] Li J, Zhao Z, Ma LS, McReynolds D, Lin Z, Chen T. (2021). Mental health among college students during the COVID-19 pandemic in China: a 2-wave longitudinal survey. J Affect Disord.

[7] Graner KM, Cerqueira ATA. (2019). Revisão integrativa: sofrimento psíquico em estudantes universitários e fatores associados. Cienc Saude Colet.

[8] Bresolin JZ, Dalmolin GL, Vasconcellos SJL, Barlem ELD, Andolhe R, Magnago TSBS. (2020). Depressive symptoms among healthcare undergraduate students. Rev Latino-Am Enfermagem.

[9] Rosa C, Nunes ES, Armstrong AC. (2021). Depressão entre estudantes de medicina no Brasil: uma revisão sistemática. Int J Educ Health.

[10] Santos NM, Santana MS, Faustino MVS, Fernandes FECV, Santos RLP. (2021). Prevalence of depression in health academic and associated factors. Braz J Dev.

[11] Bandeira M, Bekou V, Lott KS, Teixeira MA, Rocha SS. (2002). Validação transcultural do teste de orientação da vida (TOV-R). Estud Psicol (Natal).

[12] Guanilo ME, Gonçalves N, Romaniski PJ. (2019). Propriedades psicométricas de instrumentos de medidas: bases conceituais e métodos de avaliação - parte II. Texto Contexto Enferm.

[13] American Educational Research Association (AERA), American Psychological Association (APA), National Council On Measurement In Education (2014). Estándares para pruebas educativas psicológicas.

[14] Torlig EGS, Resende PC. (2019). Validação de Instrumento de Coleta de Dados: experiência com o Coeficiente de Validação de Conteúdo (CVC) e Proposição de uma nova Abordagem para Pesquisas Qualitativas Investigação Qualitativa em Ciências Sociais.

[15] Cronbach LJ. (1951). Coefficient alfa and the internal structure of tests. Psychometrika.

[16] Nunnally JC, Bernstein IH. (1994). Psychometric theory.

[17] Deng L, Chan W. (2017). Testing the Difference Between Reliability Coefficients Alfa and Omega. Educ Psychol Meas.

[18] Malkewitz CP, Schwall P, Meesters C, Hardt J. (2023). Estimating reliability: a comparison of Cronbach’s α, Mc Donald’s and the greatest lower bound. Soc Sci Human Open.

[19] Sharif AZ, Ghazi-Tabatabaei M, Hejazi M, Askarabad MH, Dehshiri GR, Sharif FR. (2011). Confirmatory Factor Analysis of the University Student Depression Inventory (USDI). Procedia Soc Behav Sci.

[20] Kaiser HF. (1974). An index of factorial simplicity. Psicometrika.

[21] Matos R. (2019). Análise Fatorial.

[22] Xia Y, Yang Y. (2019). RMSEA, CFI and TLI in structural equation modeling with ordered categorical data: the story they tell depends ont the estimation methods. Behav Res.

[23] Hu L, Bentler PM. (1999). Cutoff criteria for fit indexes in covariance structure analysis: conventional criteria versus new alternatives. Struct Eq Model.

