# Supplementary material for: University Student Depression Inventory, Brazilian Version, Construct Assessment
Source: Rev Bras Enferm. 2024 Jul 29;77(3):e20230232. doi: 10.1590/0034-7167-2023-0232 (PMC11290729; doi:10.1590/0034-7167-2023-0232)
Supplement: Translation

# Translation: Avaliação do Construto do *University Student Depression Inventory*, Versão Brasileira

**DOI:** 10.1590/0034-7167-2023-0232pt

**Authors:** Fernanda Pâmela Machado, Marcos Hirata Soares, Katya Luciane de Oliveira, Regina Celia Bueno Rezende Machado, Adriano Luiz da Costa Farinasso, Margarita Antonia Villar Luís

**Affiliations:** I Universidade Estadual de Londrina. Londrina, Paraná, Brasil; II Escola de Enfermagem de Ribeirão Preto. Ribeirão Preto. São Paulo, Brasil

**Keywords:** Estudo de Validação, Análise Fatorial, Depressão, Estudantes, Questionário de Saúde do Paciente

## Abstract

**Objetivos:**

avaliar o construto do *University Student Depression Inventory*, versão brasileira (USDI-BR).

**Métodos:**

estudo metodológico, realizado com uma amostra probabilística bola de neve constituída de 334 estudantes de graduação e pós-graduação. Foram realizadas a análise fatorial confirmatória, a confiabilidade pelo coeficiente de ômega de McDonald e o alfa de Cronbach. A análise por componentes principais foi realizada utilizando a rotação ortogonal varimax e a rotação oblíqua oblimin, por meio dos critérios de Kaiser-Meyer-Olkin, teste de esfericidade de Bartlett e *scree plot*.

**Resultados:**

o USDI-BR apresentou consistência interna dos itens de ω = 0,95 e permaneceu com 30 itens, com acréscimo de 1 fator (Desejo de morrer e retraimento social), totalizando 4 fatores.

**Conclusões:**

o USDI-BR possui evidências que remetem à sua validade e também à sua consistência interna, merecendo que novos estudos sejam realizados visando ampliar as evidências de suas propriedades psicométricas.

## INTRODUÇÃO

A depressão é um transtorno mental comum em todo o mundo. Por se tratar de uma doença com aumento significativo nos últimos anos, a Organização Mundial da Saúde (OMS) aponta que, até 2030, ela será mais prevalente em todo o planeta^(1-2)^. Devido à sua “comum” ocorrência, muitos pesquisadores realizam estudos em todo o mundo buscando ações que visem minimizar esse problema. Dois estudos destacam que a depressão apresenta quatro características principais: sintomas cognitivos; sintomas fisiológicos; sintomas comportamentais; e sintomas motivacionais^(3-4)^.

Esses sintomas podem afetar pessoas com depressão, mas, em jovens adultos, eles se intensificam diante das mudanças às quais são submetidos, principalmente aqueles na fase acadêmica. Diversas situações, como cobranças externas, autocobrança, mudanças fisiológicas e hormonais, mudanças sociais e políticas, classe social, insatisfação e sobrecarga, podem ser identificadas em jovens adultos em fase acadêmica^(1,5)^.

Além dessas mudanças, que comprometem o desempenho acadêmico e pessoal, há preocupação com os comportamentos suicidas. Atualmente, um jovem adulto que apresenta sintomas depressivos tem maior risco de comportamento suicida do que outras pessoas. Esse risco nessa população está relacionado aos domínios desta pesquisa, sendo que a motivação acadêmica e/ou sua ausência, o aspecto cognitivo/emocional e a letargia levam ao retraimento social e, consequentemente, ao desejo de morrer^(6-7)^.

Em particular, a sintomatologia depressiva associada às mudanças e cobranças exigidas pela vida acadêmica contribui para o aumento do comportamento suicida. Essa afirmação é comprovada por meio de estudos científicos realizados nessa população no Brasil, em que o jovem adulto, estudante de graduação e/ou pós-graduação apresenta fatores de risco para a sintomatologia depressiva^(8-11)^.

Devido ao aumento dos fatores de risco e da sintomatologia depressiva nessa população, é perceptível na prática clínica e na literatura o número de estudantes universitários que sofrem com problemas psíquicos. Pensando nesse aumento e, por não identificar, até o momento, na literatura brasileira, um instrumento com evidência satisfatória de validade de conteúdo específico para estudantes, dois pesquisadores^(6)^ traduziram e adaptaram para o Brasil o *University Student Depression Inventory*, versão brasileira (USDI-BR), o qual apresentou evidência satisfatória de validade de conteúdo^(6)^. Esse instrumento tem como objetivo medir o nível de sintomatologia depressiva em estudantes universitários. A escala original foi criada e validada^(4)^ por Khawaja e Bryden em 2006 na Universidade de Queensland.

No Brasil, foram realizadas a tradução e a adaptação transcultural^(6)^, ou seja, já foi realizada a validação de conteúdo. No entanto, para que esse instrumento possa ser utilizado, é necessária a validação do construto.

Pensando em responder qual o construto do USDI-BR, neste estudo, foi realizada a validação do construto por meio da aplicação do instrumento traduzido e validado na população-alvo de forma divergente, analisando se a escala está medindo o construto de modo eficiente por meio de análise fatorial confirmatória (AFC).

## OBJETIVOS

Avaliar as propriedades psicométricas do USDI-BR.

## MÉTODOS

### Aspectos éticos

A pesquisa foi aprovada pelo Comitê de Ética em Pesquisa envolvendo Seres Humanos no ano de 2020, e está em conformidade com a Resolução n°. 466 de 2012. Todos os participantes da pesquisa receberam o *link* via plataforma *Google^®^* por meio da ferramenta *Google Forms^®^* e tiveram acesso a uma cópia do Termo de Consentimento Livre e Esclarecido (TCLE).

### Desenho, local do estudo e período

Trata-se de estudo metodológico, realizado com estudantes de graduação no estado do Paraná, Brasil, no período de agosto de 2021 a janeiro de 2023.

### População, critérios de inclusão e exclusão

A amostra foi não probabilística do tipo bola de neve, e foi constituída por 334 estudantes de graduação das seguintes áreas de conhecimento: ciências exatas e da terra; ciências biológicas; engenharias; ciências da saúde; linguística; letras e artes; ciências agrárias; ciências sociais aplicadas; e ciências humanas. O critério para a participação dos estudantes na pesquisa era ter idade maior que 18 anos e estar matriculado em um curso de graduação em qualquer área de conhecimento. Foram excluídos do estudo os alunos que estavam afastados por questões de saúde.

### Protocolo do estudo

Na primeira etapa, foi levantada na literatura uma busca breve por título. A busca se deu nas bases Biblioteca Virtual de Saúde (BVS), *Scientific Electronic Library Online* (SciELO), seguindo os Descritores em Ciências da Saúde (DeCS): “Depressão”, “Estudo de validação”, “Estudantes”. Foi aplicado o filtro para os últimos cinco anos, limitado à literatura nacional para melhor elucidação da temática, porém não foi encontrado nenhuma escala voltada para estudantes universitários, apenas o estudo de adaptação transcultural do USDI-BR^(6)^.

Na segunda etapa, foram acrescentados ao *Google Forms^®^* o USDI-BR, o Teste de Orientação de Vida (TOV-R)^(12)^ e o TCLE, seguindo as normas da Comissão Nacional de Ética em Pesquisa (CONEP). O aluno entrevistado tinha a opção de baixar o TCLE em PDF e ficar com uma cópia, assim como assinalar a opção de aceite ou recusa para participar da pesquisa.

Na terceira etapa, foi encaminhado aos alunos via mídias sociais *WhatsApp* ^®^ *, Instagram* ^®^, grupos de *Facebook* ^®^, e-mail eletrônico, sendo solicitado que encaminhassem a conhecidos. Essa etapa ocorreu no período de um ano.

### Análise dos resultados e estatística

Para a validação divergente ou discriminante, utilizou-se o TOV-R, cujo objetivo dessa medida é avaliar a validade divergente do USDI-BR, ou seja, analisou a diferença entre o construto do USDI-BR e o TOV-R que, teoricamente, não apresentasse correlação. Foi empregado o coeficiente de correlação (postos) de Spearman. Trata-se de uma correlação não paramétrica^(13)^.

O TOV-R mede como as pessoas percebem suas vidas (mais otimista ou menos otimista), contendo 10 itens, divididos em positivos (itens 1, 4 e 10), negativos (itens 3, 7 e 9) e neutros (2, 5, 6 e 8). Em uma escala tipo Likert de 5 pontos, com graduações de 0 a 4, os entrevistados avaliaram o grau de concordância ou discordância em relação às questões do TOV-R. Os valores eram representados de 0 a 4, sendo que: 0 = discordo totalmente; 1 = discordo; 2 = neutro; 3 = concordo; e 4 = concordo totalmente. No Brasil, o estudo de validação apresentou consistência interna (α =0,68) satisfatória.

Para a análise fatorial exploratória (AFE) e AFC, foram inseridos e analisados pelo *software Statistical Package for the Social Sciences* (SPSS), versão 25.

Para analisar a consistência interna do USDI-BR, foi calculado o coeficiente ômega de McDonald (ω) e alfa de Cronbach (α). A análise de componente principal (ACP) foi realizada aplicando-se a rotação ortogonal varimax e rotação oblíqua oblimin após apreciação nos critérios de Kaiser-Meyer-Olkin (KMO), teste de esfericidade de Bartlett (TEB) e *scree plot*.

## RESULTADOS

### Caracterização da amostra

Participaram deste estudo 334 estudantes de nove áreas do conhecimento. Desses, 79,3% eram do sexo feminino (n=265) e 20,4% do sexo masculino (n=68). Nenhum aluno se declarou como não-binário. Em relação à faixa etária, 39,2% tinham idade entre 20 e 23 anos (n=131), 19,8%, entre 24 e 27 anos (n=66), 13,8%, entre 17 e 19 anos (n=46), 13,5%, > 30 anos (n=47), 10,8%, entre 28 e 30 anos (n=36), 40 anos (n=1) 45 anos (n=2), 52 anos (n=1), 53 anos (n=2) e 57 anos (n=2).

### Análise de confiabilidade

A análise de confiabilidade do USDI-BR foi testada por meio do coeficiente ômega de McDonald, apresentando consistência interna dos itens de ω:0,959 e alfa de Cronbach de 0,957. Para o TOV-R, também foi testada e apresentou a consistência interna de ω:0,790.

### Validade divergente

Na Tabela 1, pode-se identificar, na segunda coluna (TOV-R global), para todos os domínios, a correlação apresentando carga negativa, sugerindo que o USDI-BR tem validade divergente com o TOV-R.

**Tabela 1:** Matriz de correlação e validade divergente das escalas *University Student Depression Inventory*, versão brasileira, e Teste de Orientação de Vida, Paraná, Brasil, 2023

Escalas		TOV-R global	Letargia	Cognitivo/emocional	Motivação acadêmica
TOV-R global	Spearman	-			
	*Ρ*	-			
Letargia	Spearman	-0,448			
	*Ρ*	<0,001	-		
Cognitivo/emocional	Spearman	-0,634	0,787	-	
	*Ρ*	<,001	<0,001		
Motivação acadêmica	Spearman	-0,450	0,767	0,689	
	*Ρ*	<.001	<0,001	<0,001	-

Após a análise de confiabilidade e validade divergente, foi realizada a ACP por meio do critério de KMO e três rodadas principais, utilizando-se o método de rotação ortogonal varimax e rotação oblíqua oblimin. Foi estabelecida variância de comunalidade <0,3 para exclusão do item do instrumento.

Na primeira rodada, foram utilizadas a rotação varimax e a extração de 5 componentes. A amostra, inicialmente, apresentou KMO de 0,950 e variância explicada de 64,6%. O critério de *scree plot* (Figura 1) sugeriu o ponto de inflexão mais acentuado nos números 4 e 5, sugerindo uma escala com 4 ou 5 componentes.

**Figura 1:** *Scree plot* **dos itens do** *University Student Depression Inventory*, versão brasileira, Paraná, Brasil, 2023

Na segunda rodada, a extração foi de 4 componentes por meio da rotação oblimin. Para 4 componentes, houve queda nos valores de comunalidade de 3 fatores. A variância explicada nessa rodada foi de 61%.

Na terceira rodada, a extração foi com 3 componentes em rotação oblíqua, apresentando queda nos valores de comunalidade em 5 fatores. Quando realizada a comparação da rotação oblíqua e a varimax (ortogonal), os valores de comunalidade obtiveram queda em 10 fatores. A variância explicada em 3 componentes foi de 57,2%. Ou seja, a escala apresentaria melhor validade de construto quando disposta em 4 fatores e não os 3, como em sua construção original.

### Análise fatorial confirmatória

Nessa etapa, foram calculadas as cargas fatoriais com 3 e 4 fatores. Conforme apresentado na Tabela 2, as medidas de ajustamento não foram satisfatórias com 3 fatores. O Índice de Ajuste Corporativo (IAC) apresentou valor de 0,90 para o índice de 4 fatores, isto é, em comparação ao de 3 fatores, o de 4 fatores mostra-se um modelo mais ajustado.

**Tabela 2:** Cargas fatoriais com 3 e 4 fatores dos itens do *University Student Depression Inventory*, versão brasileira, Paraná, Brasil, 2023

Cargas Fatoriais	3 fatores	4 fatores
Índice de Ajuste Comparativo	0,847	0,905
Índice de Tucker Lewis	0,834	0,893
Raiz Quadrada Média Residual Padronizada	0,0552	0,0472
Erro Quadrado Médio de Aproximação	0,0860	0,0691
Limite superior	0,0909	0,0744
Limite inferior	0,0811	0,0639

Na Tabela 3, é possível visualizar os fatores e as descrições, assim como os itens do USDI-BR. Os itens 5 - Eu tenho pensado em me matar, 9 - Eu me pergunto se vale a pena continuar vivendo, 14 - Passo mais tempo sozinho (a) do que costumava e 28 - Ninguém se importa comigo agora, após AFE e AFC, foram acrescentados ao fator 4 - Desejo de morrer e retraimento social (DM e RS). A definição do nome do fator 4 e junção desses itens a esse fator foi realizada pelos autores deste estudo. Analisaram-se os critérios teóricos e estatísticos juntos para acréscimo ao USDI-BR.

**Tabela 3:** Domínios da escala *University Student Depression Inventory*, versão brasileira, e fatores dos itens validados pela análise fatorial confirmatória, Paraná, Brasil, 2023

Fator	Descrição	Itens (t=30)^*^
Letargia	Caracterizado por uma combinação de manifestações fisiológicas, comportamentais e cognitivas.	1,2,3,4,8,10,29,30
Cognitivo/emocional	Representa os sintomas cognitivos, sintomas emocionais.	6,11,18,20,21,22,23,24,25,26
Motivação acadêmica	É caracterizado pela motivação e procrastinação relacionado ao estudo.	7,12,13,15,16,17,19,27
Desejo de morrer e retraimento social	Representado pelo isolamento social e sentimentos de morte.	5,9,14,28

* *Total de itens da escala USDI-BR.*

Na Figura 2, encontra-se o modelo testado pela AFC. Nele, é notório o acréscimo de mais um fator, conforme havia sido sugerido na análise exploratória. Essa distribuição e esse acréscimo de mais um fator apresentaram valores de comunalidades significativos entre os itens, diferente do que havia observado anteriormente, quando o USDI-BR continha 3 fatores.

**Figura 2:** Modelo teórico selecionado, testado e validado pela análise fatorial confirmatória conforme os fatores do *University Student Depression Inventory*, versão brasileira, Paraná, Brasil, 2023

O valor de *p* dos itens após transferência para o domínio 4 obteve resultados significativos, ou seja, todos os itens, incluindo os itens 5 (*Ρ*=0,72), 9 (*Ρ*=,81), 14 (*Ρ*=0,74) e 28 (*Ρ*=76)), apresentaram correlação significativa.

Além do modelo teórico, pode-se analisar, por meio dos dados, o quão relacionados estão os fatores cognitivos/emocionais com a letargia, a motivação ou desmotivação no âmbito acadêmico, assim como o isolamento social com o sentimento de querer morrer de estudantes de graduação e pós-graduação.

O menor valor está relacionado ao fator 2 (item 18 - Eu me sinto tímido(a) quando estou com outras pessoas (*p*=0,48)). O maior valor foi em relação ao fator 3 (item 15 - Eu não acho os estudos tão interessantes quanto eram (*p*=0*,84*)).

## DISCUSSÃO

Para testar a validade de medida de um instrumento, estudos como os realizados pela *American Eduactional, Research Association* (AERA) e *American Psychological Association* (APA)^(13-14)^ consideram a validade de conteúdo, validade de critério e validade de construto como as três técnicas principais para essa finalidade.

No USDI-BR, realizou-se a validade de conteúdo para a versão brasileira^(6)^, apresentando Coeficiente de Validade de Conteúdo total (CVCt) de 0,94, ou seja, um valor considerado adequado para um instrumento de medida que deve ser igual ou maior a 0,80^(14)^.

Para validar e dar continuidade em todas as etapas preconizadas no processo de validação, este estudo realizou a validade de construto com uma amostra de 334 estudantes. Para este fim, foram utilizado dois coeficientes (ômega de McDonald e alfa de Cronbach), e ambos realizaram a mesma função: medir a confiabilidade de um instrumento^(15-17)^.

A confiabilidade do instrumento por meio da análise do ômega de McDonald apresentou consistência interna dos itens de ω=0,95 e alfa de Cronbach de α=0,95, ou seja, valor que representa confiabilidade alta, pois valores iguais ou maiores a 0,70 são considerados adequados em relação ao construto^(18)^. Outros estudos realizados com o *University Student Depression Inventory* (USDI) apresentaram valores próximos ao do presente estudo, como a versão no idioma português de Portugal (α= 0,80), persa do Irã (α= 0,94)^(19)^ e a versão original, inglês da Austrália (α= 0,95)^(4)^, corroborando o que foi identificado no USDI-BR.

Para a validade divergente, foi utilizada a correlação de Spearman, podendo identificar, por meio deste, que os valores encontrados na matriz de correlação (Tabela 1) evidenciaram valores negativos. Os valores negativos apresentaram baixas correlações em relação ao TOV-R e USDI-BR, isto é, o TOV-R tem como função medir as expectativas, os otimismo e eventos futuros em relação à vida, apresentando, neste estudo, baixas correlações em relação a todos os fatores do USDI-BR, principalmente ao fator motivação acadêmica (ρ= -0,450)^(13)^.

A rotação ortogonal dos fatores, por meio do método varimax (fatores não são correlacionados) e rotação oblíqua oblimin (fatores correlacionados), assim como os critérios de KMO, TEB e *scree plot* (Figura 1), sugeriu um modelo, *a posteriori*, onde a comunalidade entre os componentes analisados sugeriu o acréscimo de mais um fator ao USDI-BR.

Após as hipóteses sugestivas para adequar mais um fator ao USDI-BR, um dos testes realizados para verificar a adequabilidade de uma amostra é o KMO. Os fatores referenciais mais próximos a 1 condizem com dados ajustados à análise fatorial (AF)^(20-22)^. No presente estudo, o KMO foi muito significativo, indicando AF ajustado (KMO= 0,95). O estudo realizado no Irã se assemelha ao valor identificado neste estudo (KMO= 0,94)^(19)^. Ambos os estudos estão dentro da classificação de KMO com adequabilidade da amostra excelente.

O Índice de Ajuste Comparativo (CFI) na versão original, no idioma inglês da Austrália, foi > 0,90^(4)^. No estudo atual, o CFI com 4 fatores foi de 0,90, e com 3 fatores, 0,84. O CFI compara o modelo base e calcula o ajuste relativo ao modelo. O valor de referência considerado “ótimo ajuste ao modelo” é considerado de 0,9. Ou seja, abaixo de 0,90 indica ajuste adequado^(21-23)^. O USDI-BR encontra-se dentro dos parâmetros adequados.

Em relação ao Índice de Tucker Lewis (TLI), neste estudo, o valor foi de 0,83 para a carga de 3 fatores e 0,89 para a carga de 4 fatores, ou seja, não se enquadram nos valores considerados como “ótimo ajuste”, que seriam de 0,90 a 0,95, visto que o TLI visa avaliar os ajustes relativos do modelo ao compará-lo a outro modelo^(23)^. Assim seja, o ajuste em relação ao TLI, no presente estudo, demonstrou-se abaixo do considerado ideal, contudo, uma melhoria significativa foi alcançada no modelo de 4 componentes.

A Raiz Quadrada Média Residual (SRMR) padronizada também sugeriu que o modelo forneceu um bom ajuste aos dados (SRMR= 0,086), e o estudo realizado no Irã o SRMR foi de 0,077^(19)^. Para fortalecer esses dados, o Erro Quadrado Médio de Aproximação (RMSEA) deve variar de 0 a 1, porém, valores menores ou próximos a 0,6 sugerem modelos mais ajustados. Neste estudo, o RMSEA foi testado com 3 (RMSEA= 0,086) e 4 fatores (RMSEA= 0,069), dentro do valor limite, para que seja considerado um modelo bem ajustado, conforme esse critério.

Os modelos testados demostraram que o mais indicado seria uma escala com um fator 4, corroborando o que já havia sido pontuado pelos juízes na versão traduzida e adaptada para a versão brasileira do USDI^(6)^. Sendo assim, o USDI-BR permanece com 30 itens, porém com 4 fatores. O fator 1 refere-se à letargia, 2, ao cognitivo/emocional, 3, à motivação acadêmica, e 4, ao desejo de morrer e retraimento social. O fator 4 distribuiu os valores de comunalidades mantendo a sua distribuição esperada. Apenas o item 18 apresentou carga negativa.

No que se refere ao domínio cognitivo/emocional, o item 18 (Eu me sinto tímido(a) quando estou com outras pessoas) tem cargas relativamente baixas em seu fator, corroborando o estudo de AF do estudo original, no qual os autores da escala original sugerem que esse item demonstra uma medida suspeita e deve ser melhor investigado nos estudos futuros^(4)^. Esse achado deve ser levado em consideração para a revisão contínua das propriedades psicométricas de um instrumento de medida. Uma escala pode deixar de ser válida para uma população decorrente do tempo, das mudanças culturais, termos e palavras em desuso, tornando-se não mais viável para aquela cultura.

### Limitações do estudo

O USDI-BR apresentou resultados promissores na AFC. Contudo, é importante notar algumas limitações no estudo. Primeiro, o número de entrevistados foi relativamente baixo, o que sugere a necessidade de uma amostra maior que inclua estudantes de diferentes estados brasileiros, permitindo uma adaptação mais precisa às nuances culturais de cada região. Além disso, a predominância de participantes do sexo feminino na pesquisa pode requerer ajustes no desenho amostral para garantir uma representatividade equilibrada.

### Contribuições para a área da enfermagem, saúde ou política pública

A escala apresenta boa consistência interna, além de ser, até o presente, a única validada no Brasil para estudantes universitários. O USDI-BR possibilitará que profissionais de saúde e professores utilizem um instrumento que possa ser eficaz na mensuração dos sintomas depressivos em estudantes universitários. Posto isso, fomenta-se que uma escala como essa auxiliaria no rastreio dos sintomas, bem como com um investimento apropriado poderia ser desenvolvido de modo a atender aos profissionais de serviços de atenção à saúde mental.

## CONCLUSÕES

Este estudo demonstrou, por meio da AF, que o USDI-BR apresentou evidência de validade de conteúdo satisfatória. Mantiveram-se os 30 itens, porém foi acrescido mais um fator, totalizando 4 fatores com boa evidência de validade do construto.

Sugere-se, para os próximos estudos, testar outras propriedades psicométricas do USDI-BR, como a validade preditiva e validade discriminante. É recomendado que sejam realizadas pesquisas com estudantes de graduação e pós-graduação de todo o território brasileiro aplicando o USDI-BR, levando em consideração a diversidade cultural que existe no Brasil.

Além da aplicabilidade do instrumento, recomenda-se que sejam investidos em outros meios que visem à prevenção dos casos (educação continuada das equipes multiprofissionais, educação em saúde nas escolas e universidades), desenvolvimento de materiais educativos (*e-books* e *folders*) e divulgação nas mídias sociais. Além desses, é relevante fortalecer as políticas públicas em saúde mental, como moradia, renda, trabalho, educação e assistência social.
